# Revealing Cryptic Changes of Cyanobacterial Community Structure in Two Eutrophic Lakes Using eDNA Sequencing

**DOI:** 10.3390/ijerph17176356

**Published:** 2020-09-01

**Authors:** Yongguang Jiang, Peng Xiao, Gongliang Yu, Gaofei Song, Renhui Li

**Affiliations:** 1 Department of Biological Sciences and Technology, School of Environmental Studies, China University of Geosciences, Wuhan 430074, China; jiangyg@cug.edu.cn; 2 Aquatic Ecohealth Group, Key Laboratory of Urban Environment and Health, Institute of Urban Environment, Chinese Academy of Sciences, Xiamen 361021, China; 3 Key Laboratory of Algal Biology, Institute of Hydrobiology, Chinese Academy of Sciences, Wuhan 430072, China; yugl@ihb.ac.cn (G.Y.); song@ihb.ac.cn (G.S.); reli@ihb.ac.cn (R.L.)

**Keywords:** eutrophication, cyanobacterial community, *Microcystis*, diversity, eDNA

## Abstract

Harmful cyanobacterial blooms pose a risk to human health worldwide. To enhance understanding on the bloom-forming mechanism, the spatiotemporal changes in cyanobacterial diversity and composition in two eutrophic lakes (Erhai Lake and Lushui Reservoir) of China were investigated from 2010 to 2011 by high-throughput sequencing of environmental DNA. For each sample, 118 to 260 *cpcBA*-IGS operational taxonomic units (OTUs) were obtained. Fifty-two abundant OTUs were identified, which made up 95.2% of the total sequences and were clustered into nine cyanobacterial groups. Although the cyanobacterial communities of both lakes were mainly dominated by *Microcystis*, Erhai Lake had a higher cyanobacterial diversity. The abundance of mixed Nostocales species was lower than that of *Microcystis*, whereas *Phormidium* and *Synechococcus* were opportunistically dominant. The correlation between the occurrence frequency and relative abundance of OTUs was poorly fitted by the Sloan neutral model. Deterministic processes such as phosphorus availability were shown to have significant effects on the cyanobacterial community structure in Erhai Lake. In summary, the *Microcystis*-dominated cyanobacterial community was mainly affected by the deterministic process. Opportunistically dominant species have the potential to replace *Microcystis* and form blooms in eutrophic lakes, indicating the necessity to monitor these species for drinking water safety.

## 1. Introduction

Cyanobacterial blooms in eutrophic freshwater bodies are a globally severe environmental problem [1,2]. The massive proliferation of cyanobacteria has increased the difficulty and cost of water management of urban water supplies [3]. Off-flavor compounds are released from both growing and decaying blooms, resulting in problems with undesirable odors in ambient air and drinking water [4,5]. Moreover, the oxidation of large amounts of organic matter released from cyanobacterial cells may cause oxygen depletion, which is lethal to many aquatic animals such as fish [6]. Harmful cyanobacteria can also produce hepatotoxins, cytotoxins, neurotoxins, and dermatotoxins [7]. Some cyanotoxins are resistant to degradation and persist for a long time in natural environments [8]. As a result, cyanobacterial blooms and their metabolites pose a great risk to the health of aquatic ecosystems and drinking water safety. Therefore, it is important to monitor the dynamics of cyanobacterial communities in water bodies in order to manage cyanobacterial blooms in good time.

Generally, freshwater cyanobacterial blooms are caused by *Microcystis*, *Dolichospermum*, *Aphanizomenon*, *Raphidiopsis*, and *Planktothrix*, and these genera are characterized by different toxicities. Moreover, each of above genera contains both toxin-producing and nontoxin-producing species. *Microcystis* is the most frequently reported bloom-forming cyanobacterium, which often occurs as dense blooms in summer and autumn [9,10,11]. In subtropical and temperate lakes, *Dolichospermum* and *Aphanizomenon* usually become dominant when the abundance of *Microcystis* decreases in winter and spring [12,13,14]. However, *Raphidiopsis* forms perennial blooms in many tropical and subtropical lakes [15], whereas *Planktothrix* can be exclusively dominant in temperate lakes with relatively low water temperatures [16,17]. Therefore, temperature is a critical factor affecting the composition of the cyanobacterial community.

Global warming is predicted to enhance the expansion of cyanobacterial blooms [18], and it is also a catalyst for the alteration of the cyanobacterial community structure. For example, *Raphidiopsis* has already invaded temperate lakes and become predominant in some of them [19,20]. In addition to temperature, other factors such as nutrient salts, carbon dioxide, and light intensity also contribute to the competition and succession of bloom-forming cyanobacteria [15,18,21,22,23]. However, these studies are mainly based on the morphological identification of cyanobacterial species. Information on genetic differences within the same cyanobacterial populations and communities is lacking.

Previous studies have revealed that populations of bloom-forming cyanobacteria are highly diverse [9,24,25]. During a bloom, dynamic changes occur in the genotypic composition of a cyanobacterial population [11,24,25]. In a previous study, the composition of *Microcystis* genotypes and morphotypes varied periodically and were significantly affected by water temperature [11]. The conditions favorable for *Microcystis* growth are more beneficial for nontoxin-producing genotypes, whereas unfavorable growth conditions can increase the relative abundance of toxin-producing genotypes [24]. Moreover, it has been demonstrated that the proportion of toxin-producing genotypes of *Microcystis* is mainly affected by water temperature and phosphate concentration [11]. The genotypic succession of *Dolichospermum* was also found to be triggered by water temperature in a hyper-eutrophic lake [25]. These results indicate that there are various genotypes adaptive to different environments within the same cyanobacterial population. Therefore, the genetic characterization of cyanobacterial blooms could enhance our understanding of the biological mechanism of bloom formation.

So far, the majority of studies have focused on the aforementioned dominant bloom-forming species. Moderately abundant and rare species are often ignored, and few researches have focused on their potential to become more prominent in the cyanobacterial community. However, some cyanobacterial species are opportunistically dominant and may be exclusively dominant under appropriate environmental conditions [26,27]. Species-specific responses to global warming may also increase the frequency of massive proliferation of rare species [28,29]. To predict bloom development accurately, more research on the genetic diversity and dynamics of the whole cyanobacterial community is needed [30]. In this study, two eutrophic freshwater lakes (Erhai Lake and Lushui Reservoir in China) with annual *Microcystis* blooms were monitored to systematically characterize the dynamic changes in the genetic diversity and composition of the cyanobacterial community from 2010 to 2011. Both surface and deep water were investigated to provide information about cyanobacterial distribution in the whole water column.

## 2. Materials and Methods

### 2.1. Sampling Sites and Collection of Cyanobacteria

**Figure 1:**
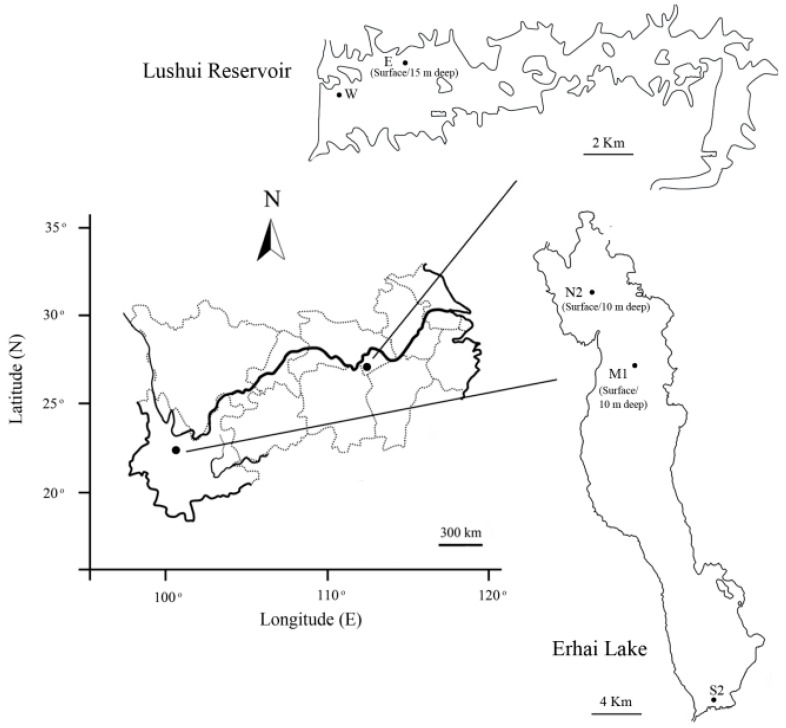
Map of Erhai Lake and Lushui Reservoir showing sampling locations in this study.

Erhai Lake is a plateau freshwater lake in southwestern China (25°36′–25°54′ N, 100°06′–100°18′ E; water area = 249 km^2^; average depth = 10.2 m; maximum depth = 20.7 m). Lushui Reservoir is a freshwater lake in central China (29°39′–29°42′ N, 113°53′–114°03′ E; water area = 57 km^2^; average depth = 8.4 m; maximum depth = 26.4 m). Both Erhai Lake and Lushui Reservoir are located in the subtropical monsoon climate zone, and they have been suffering from eutrophication and annual *Microcystis* blooms. Water samples from Erhai Lake were collected in 2010, as described previously [31]. The surface (0–0.5 m) water from three sites (N2, M1, and S2) in four months throughout the year (February, May, August, and December) were used in this study. Water samples at a depth of 10 m were collected from the N2 and M1 sites at the same time. Water samples from Lushui Reservoir were collected at two sites (W and E) in three months (February, May, and September) of 2011. The surface water from the two sites and water samples at a depth of 15 m from the E site were used. In total, 28 water samples were collected. All the samples were numbered according to sampling sites, depth, and month, and their information is listed in Figure 1 and Table S1.

For each sample, cyanobacteria were collected by filtering a volume of 300 mL water through a polycarbonate membrane filter (0.45 μm pore size, Millipore, USA). The filters were preserved at −80 °C prior to DNA extraction. Surface water samples were also used to analyze the concentrations of total phosphorus (TP), total nitrogen (TN), ammonium nitrogen (NH_4_-N), and nitrate nitrogen (NO_3_-N), as described by Wu et al. (2006) [32]. The water temperature (WT) and dissolved oxygen (DO) were measured using YSI PH100 [31], and the water transparency (SD) was measured using a Secchi disk.

### 2.2. High-Throughput Sequencing of Cyanobacterial cpcBA-IGS

Environmental DNA (eDNA) was extracted from the filters using the method developed in an earlier study [33]. Cyanobacterial *cpcBA*-IGS sequences were amplified from the eDNA of all the samples using a forward primer, PCβF [34], targeting the *cpcB* gene, and a reverse degenerate primer, PCαR454 [26] targeting the 5′ end of the *cpcA* gene. Adapters and specific barcode sequences for each sample were designed and added to the 5′ side of the PCαR454 primer for 454 pyrosequencing (Table S2, in the supplementary data). As described by Jiang et al. (2017) [26], PCR reactions were performed in triplicate using Tks Gflex DNA polymerase (Takara, Japan), and the amplification products were purified and pooled to minimize random bias. High-throughput sequencing was performed using the GS-FLX Titanium platform (Roche 454 Life Sciences, Branford, CT, USA). High-quality reads were selected by discarding low-quality reads, reads with barcode and/or primer errors, sequences shorter than 200 bp, and chimeras. Afterwards, robust sequences were obtained by trimming the barcode and primer sequences from the 5′ end. The sequence data were deposited under the accession number PRJNA638599 in the Sequence Read Archive of National Center for Biotechnology Information (NCBI).

### 2.3. Sequence Clustering

Unique sequences were picked out from the dataset of robust sequences, followed by aligning against an alignment template of reference cyanobacterial sequences [26] using Mothur v.1.33.0 [35]. The aligned *cpcBA*-IGS sequences were clustered into operational taxonomic units (OTUs) using the average neighbor clustering algorithm with a distance of 0.05 by Usearch v11.0.667 [36]. Prior to further analysis, the sequences of all the samples were subsampled and normalized to the smallest read number (i.e., 4560 in EHM11002). Subsequently, the OTU richness, Coverage, Chao 1 index, Shannon–Wiener index (here called Shannon index), Simpson index, Simpson’s evenness index, and rarefaction values were estimated using the vegan package of R v3.6.2 (https://www.r-project.org).

### 2.4. Taxonomy Assignment of OTUs

Representative sequences of OTUs were aligned with *cpcBA*-IGS sequences from the reference cyanobacterial strains used by Jiang et al. (2017) [26]. The alignment was conducted using the ClustalW v2.0 program (Conway institute, University College Dublin, Ireland). A phylogenetic tree of *cpcBA*-IGS sequences was created based on the neighbor-joining algorithm using the MEGA v7.0 software [37]. Nucleotide substitution was fitted by the Kimura 2-parameter model, and the bootstrap replications were set as 1000. The OTUs were annotated by the closest related cyanobacterial species on the phylogenetic tree. The taxonomy assignment of each OTU was also verified by homologous searching against the GenBank database on the NCBI website.

### 2.5. Neutral Model for Cyanobacterial Community

The impacts of stochastic and deterministic processes on the assembly of cyanobacterial communities were evaluated by predicting the relationship between the occurrence frequency and relative abundance of OTUs using the Sloan neutral model [38,39]. Subsequently, the OTU data of Erhai Lake and Lushui Reservoir were fitted to the neutral model. Two parameters, the goodness of fit (R^2^) and immigration rate (*m*), were calculated. The 95% confidence intervals around the fitting statistics were estimated by bootstrapping, and the bootstrap replicates were set as 1000. All model calculations were performed using R v3.6.2.

### 2.6. Statistical Analysis

The OTU abundance was the square root transformed and used for calculating the Bray–Curtis and Jaccard dissimilarity among samples in each water body. The independent effects of different environmental variables on the beta diversity of samples were estimated by hierarchical partitioning [40] and statistical significance was calculated by a randomization test. The correlations between environmental factors and cyanobacterial community structure were also evaluated by a Mantel test [41] using Pearson’s coefficients. The environmental factors were ln (x + 1) transformed and Euclidean distances between samples were calculated. The effects of the ratio of TN and TP (TN/TP) on cyanobacterial community structure was also evaluated by a Mantel test. All analyses were performed using the hier.part and vegan packages of R v3.6.2.

## 3. Results

### 3.1. Sequence Data of cpcBA-IGS

Twenty-eight filter samples were collected from Erhai Lake and Lushui Reservoir, respectively (Table S1). High-quality eDNA was extracted from the filters, and *cpcBA*-IGS was amplified and sequenced. A total of 210,806 robust *cpcBA*-IGS reads were obtained after sequence processing. Each sample comprised 4560 to 11,784 sequences. The length of all the *cpcBA*-IGS reads varied from 267 bp to 418 bp (Figure S1 in the supplementary data), indicating a high variability of this genomic region.

### 3.2. OTU Composition and Diversity

Following the normalization of the sequence number for each sample, a total of 3339 OTUs were obtained with 118 to 260 OTUs per sample (Table 1). Overall, the samples from Erhai Lake contained more OTUs than those from Lushui Reservoir. For all the samples, a relatively low coverage of OTU richness was found, with values between 0.94 and 0.97. The Chao 1 index values were much higher than the OTU richness values, suggesting the existence of many rare OTUs. This result was consistent with the extremely low evenness (≤0.03) of the OTU composition. The rarefaction curves of the OTU richness and Chao 1 index did not plateau at the sequencing depth in this study (Figure S2A,B), indicating that there were undetected OTUs, as suggested by the low coverage of OTU richness.

The Shannon index (H′) and Simpson’s reciprocal index (D^−1^) were calculated based on the OTU composition of each sample (Table 1). These two diversity indices varied similarly among the samples. In Erhai Lake, although the highest OTU diversity was found in one December sample, EHN2D1012 (OTU = 260, H′ = 3.00, D^−1^ = 8.90), other samples collected in December always had a lower OTU diversity despite being from different sampling sites. For both water bodies, the OTU diversity of each site was relatively high in February, with the exception of the S2 site in Erhai Lake. The rarefaction curves of the Shannon index were in close proximity to the plateau (Figure S2C, in the supplementary data), indicating that most of the abundant OTUs had been detected, as the Shannon index is mainly affected by the percentage of abundant OTUs.

### 3.3. Cyanobacterial Community Structure

The normalized abundance of each OTU was calculated. Subsequently, the OTUs with an abundance accounting for more than 1% of the 4560 in the whole dataset were selected and defined as abundant OTUs. Fifty-two abundant OTUs accounted for 95.2% of the total sequences (Additional file 1). Therefore, these OTUs represented the majority of the cyanobacterial community. Through a phylogenetic analysis, the 52 OTUs were classified into nine groups of cyanobacteria, including *Synechococcus* group I (11 OTUs), *Synechococcus* group II (9 OTUs), *Synechococcus*-related species (6 OTUs), *Synechocystis* (2 OTUs), *Microcystis* (12 OTUs), *Raphidiopsis* (1 OTU), mixed Nostocales species (7 OTUs), *Planktothrix* (2 OTUs), and *Phormidium* (2 OTUs) (Figure S3, in the supplementary data). The mixed Nostocales species were composed of *Dolichospermum*, *Anabaena*, *Aphanizomenon*, and *Cuspidothrix*, which could not be discriminated in the phylogenetic tree of this study.

**Figure 2:**
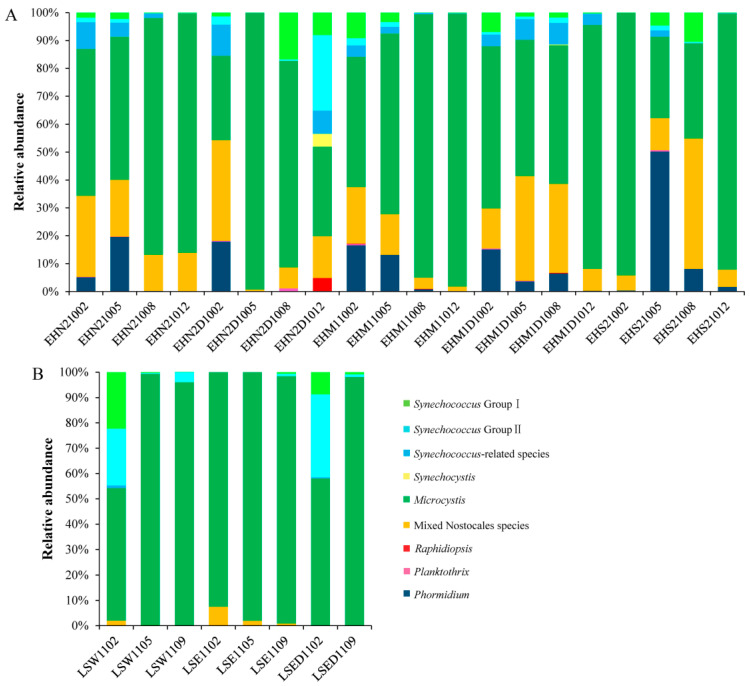
Succession of the cyanobacterial communities in Erhai Lake (**A**) and Lushui Reservoir (**B**). Only the 52 most abundant OTUs (OTU size ≥ 45) are shown.

As displayed in Figure 2, *Microcystis* was the most abundant cyanobacterial genus in both water bodies. The relative abundance of *Microcystis* varied between 29 and 99% in Erhai Lake and between 52 and 99% in Lushui Reservoir. In Erhai Lake, mixed Nostocales species were also dominant and their relative abundance even exceeded *Microcystis* in two samples, EHN2D1002 and EHS21008, with 36 and 47% mixed Nostocales species, respectively. *Phormidium* was an opportunistically dominant species in Erhai Lake, with an abundance of 50% in the EHS21005 sample. The predominance of *Synechococcus* group II only occurred in the EHN2D1012 sample with a relative abundance of 27%. However, in Lushui Reservoir, a high relative abundance of *Synechococcus* group II existed in two samples, 22 and 33% in LSW1102 and LSED1102, respectively. *Synechococcus* group I was dominant in LSW1102, with a relative abundance of 22%.

**Figure 3:**
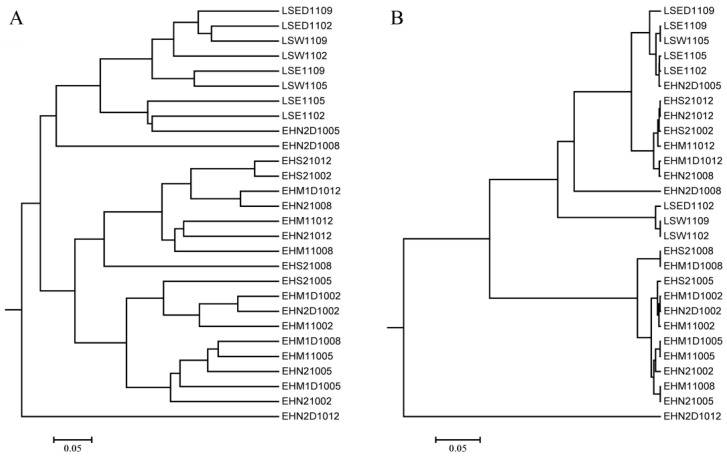
Cluster dendrograms based on membership (Jclass, **A**) and structure (Jabund, **B**) of the cyanobacterial community.

The Jaccard dissimilarities among samples were calculated and used to construct cluster dendrograms on the basis of shared abundant OTUs. According to the presence of OTUs, the samples from different water bodies tended to form independent clusters, with the exception of the EHN2D1005 and EHN2D1008 samples, which were closely related to the samples from Lushui Reservoir (Figure 3A). However, several samples from Erhai Lake were clustered together with the samples from Lushui Reservoir based on the abundance of OTUs (Figure 3B). The divergence of cyanobacterial communities at different depths was site- and month-dependent. The surface and deep water samples were obviously different for the N2 site in Erhai Lake in May, August, and December. In each water body, seasonal differences were not obvious for both the membership and structure of the cyanobacterial community.

### 3.4. Application of the Neutral Model for Cyanobacterial Community

**Figure 4:**
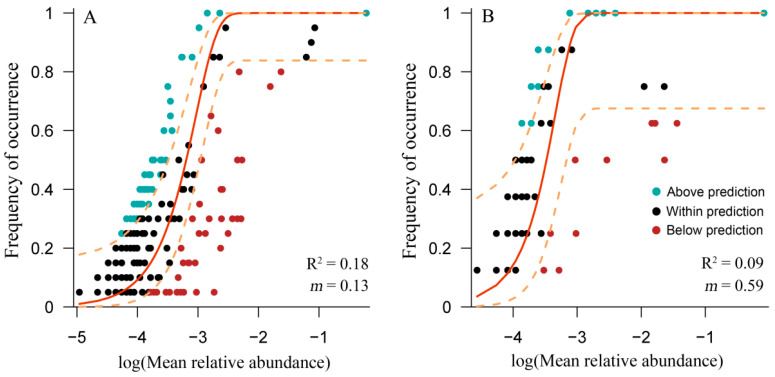
Neutral model simulation of cyanobacterial communities from Erhai Lake (**A**) and Lushui Reservoir (**B**). The solid lines indicate the best-fit theoretical description of the OTU assembly for each water body. Dashed lines represent the 95% confidence intervals for the model prediction.

The Sloan neutral model describes the stochastic assembly of the microbial community. For the relationship between the OTU occurrence frequency and relative abundance in Erhai Lake and Lushui Reservoir, the values of R^2^ and *m* of the neutral model were low (Figure 4). These results indicated that the stochastic process was not the main factor driving the assembly of cyanobacterial communities in these two water bodies. Moreover, the abundant OTUs, with greater abundances than that predicted by the neutral model, belonged to *Microcystis* in both water bodies.

### 3.5. Correlations Between Environmental Factors and Cyanobacterial Community

**Figure 5:**
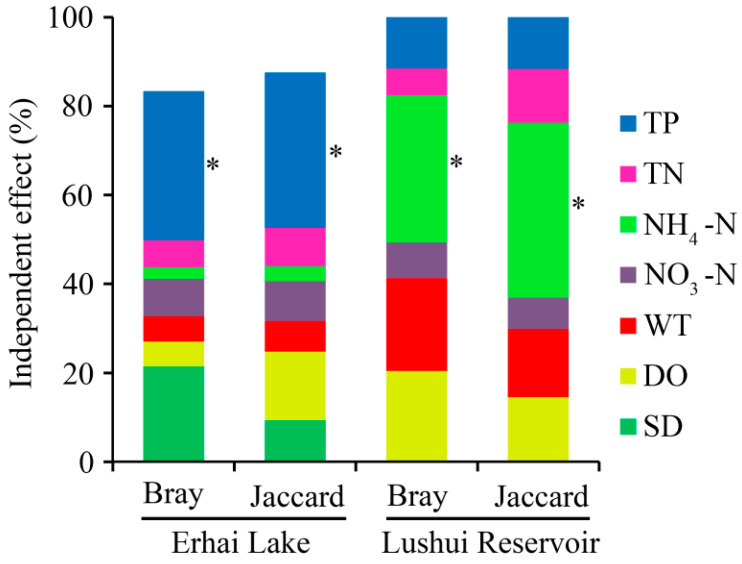
Independent effects of environmental factors on community dissimilarity as estimated from hierarchical partitioning. Asterisks indicate significant effects (*p* < 0.05, randomization test). SD values for the Lushui Reservoir were not available.

As shown in Figure 5, the TP had significant (*p* < 0.05, randomization test) independent effects for both Bray–Curtis and Jaccard dissimilarities of the cyanobacterial community in Erhai Lake. In addition, the effects of TP were larger than those of TN, NH_4_-N, NO_3_-N, WT, DO, and SD. For the cyanobacterial community in Lushui Reservoir, NH_4_-N had significant (*p* < 0.05, randomization test) independent effects for their dissimilarities, and the effects were larger than those of TP, TN, NO_3_-N, WT, and DO.

The results of the Mantel test revealed that TN/TP had significantly (*p <* 0.01, Mantel test) effects on the cyanobacterial community composition in Erhai Lake (Table 2), whereas the effects of TP was relatively weak (0.05 < *p <* 0.10, Mantel test). The cyanobacterial community composition in Lushui Reservoir was correlated (*p* ≤ 0.05, Mantel test) with the WT and had a weak correlation with the DO (0.05 < *p <* 0.10, Mantel test).

## 4. Discussion

In this study, the diversity and composition of cyanobacterial communities in two eutrophic freshwater lakes were described using OTUs obtained based on the results of next-generation sequencing. Correlations between OTU occurrence and abundance were tested using the Sloan neutral model. The effects of environmental factors on cyanobacterial community structure were also evaluated.

The results of this study revealed that the two eutrophic lakes have different cyanobacterial community structures, although they both displayed *Microcystis* blooms. Compared to Lushui Reservoir, a larger cyanobacterial diversity was observed in Erhai Lake. In a previous investigation, an extremely large number of genotypes were detected for *Microcystis* in Erhai Lake [9]. A negative correlation was determined between the *Microcystis* diversity and eutrophication levels [9]. These findings were verified in the present study because the eutrophication level was lower in Erhai Lake than in Lushui Reservoir, which had higher nutrient concentrations (Table S3 in the supplementary data). Therefore, it seems that the genetic diversity of both cyanobacterial communities and populations of individual species were affected by the eutrophication levels.

In most cases, *Microcystis* had a relatively higher abundance than mixed Nostocales species, although the latter also occurred throughout the year. Earlier studies found that predation by zooplankton is a key factor controlling the relative abundance of different cyanobacterial species in aquatic ecosystems [42,43]. Compared to the filaments of Nostocales species, *Microcystis* colonies are more resistant to grazers [42,43]. In addition, there are interspecific interactions, such as allelopathic effects between *Microcystis* and Nostocales species. Under nutrient-replete conditions, allelochemicals released from *Microcystis* can suppress the nitrogen fixation of *Dolichospermum* and lead to cytotoxicity [21]. In co-culture experiments, the growth of *Aphanizomenon* was inhibited by some *Microcystis* strains, and this effect was caused by inducible unknown chemicals secreted by *Microcystis* [44]. A previous study also found that biological interactions probably contribute to a large proportion of the inter-annual variability of cyanobacterial bloom [23]. Therefore, the cyanobacterial community structure is influenced by both biological interactions and environmental factors [22].

The difference of cyanobacterial community structure between surface and deep water was site- and month-dependent. *Microcystis*, mixed Nostocales species, *Synechococcus*, and *Phormidium* constituted the major portion of the cyanobacterial community from surface to deep water in Erhai Lake, whereas *Phormidium* was scarce in some samples. However, *Microcystis* and *Synechococcus* dominated the cyanobacterial community throughout the water column in Lushui Reservoir. These results are in contrast with previous findings in another deep lake, Dongzhen Reservoir, where distinct cyanobacterial community structures were found between the surface and deep water [26]. *Synechococcus* was completely dominant in the surface water of Dongzhen Reservoir [26]. The proliferation of *Synechococcus* was also recorded in other freshwater lakes [27] and lagoons [45]. Due to the tiny size of this cyanobacterium, little information is available about the ecological risk of its blooms. However, *Synechococcus* blooms may be lethal to sponges and seagrass, and may have a long-term impact on aquatic ecosystems by increasing baseline chlorophyll *a* concentrations [45].

As an opportunistically dominant species, *Phormidium* proliferated occasionally in Erhai Lake. However, reports on bloom-forming *Phormidium* are scarce. In the 1980s, *Phormidium* blooms, which damaged tap water by releasing off-flavor compounds, occurred annually in a deep lake (Biwa Lake in Japan) [46]. *Phormidium* was also one of the dominant cyanobacterial genera causing cyanobacterial blooms in the Greater Sudbury Area lakes of Canada [47]. However, the global proliferation of *Phormidium* is rising in rivers, where it forms benthic mats [48]. In addition, several species of *Phormidium* produce neurotoxic anatoxins [49]. Therefore, it is also necessary to monitor the abundance of *Phormidium* and its toxin content in freshwater lakes where this species is known to occur frequently.

Results from the Sloan neutral model suggested that the assembly of cyanobacterial communities in the two eutrophic lakes was affected by both stochastic and deterministic processes, and the latter may play a more important role. According to the results of hierarchical partitioning and the Mantel test, the cyanobacterial community composition of Erhai Lake was correlated with the TP. Previous studies have also suggested the key role of phosphorus in the succession and dominance of different cyanobacterial populations [11,21,22,28]. Thus, phosphorus concentration is likely to be a deterministic factor of the cyanobacterial community structure. For Lushui Reservoir, the results of hierarchical partitioning and the Mantel test were not consistent and significant environmental factors affecting the cyanobacterial community were not determined. Compared to TP (0.02 ± 0.01 mg L^−1^) in Erhai Lake, the TP (0.03 ± 0.01 mg L^−1^) in Lushui Reservoir was higher, indicating that phosphorus may not be a limiting factor for cyanobacteria in this water body. Moreover, in Lushui Reservoir, the OTU diversity was relatively low and the OTU01 of *Microcystis* had an extremely high relative abundance of 88–95% in most of the samples, indicating a robust cyanobacterial community structure dominated by *Microcystis*.

According to the TN/TP criteria [50], both Erhai Lake and Lushui Reservoir had high TN/TP values (Table S3) and seemed to be phosphorus-limited (TN/TP > 17). However, a significant correlation between the cyanobacterial community composition and TN/TP was observed in Erhai Lake but not Lushui Reservoir. This result may be ascribed to the higher nutrient concentrations in Lushui Reservoir. A previous study also suggested that TN/TP was an effective environmental factor for cyanobacterial bloom only within a certain nutrient level [31].

In addition, our findings revealed that only abundant OTUs belonging to *Microcystis* were found to have occurrence frequencies above the neutral model prediction. The dominance of *Microcystis* across the year was consistent with the strong acclimation of this species to different water temperatures [51,52]. In terms of the life-cycle of *Microcystis*, early studies proposed that this cyanobacterium overwinters in the sediment [53,54,55,56]. Recently, in a shallow eutrophic lake, a *Microcystis* bloom was found to persist in winter although at a relatively lower abundance than in summer [52]. The phenotypic plasticity of *Microcystis* in response to variations in CO_2_ concentration could also be beneficial for the adaptation of this species to different environments [57]. Thus, the occurrence and strength of *Microcystis* blooms will probably be intensified under the conditions of climate warming and elevated CO_2_ concentrations [22,57,58].

## 5. Conclusions

A similar cyanobacterial community structure may distribute across the water column of eutrophic lakes. The diversity and composition of cyanobacterial communities are affected by the geochemical characteristics of individual water bodies. Deterministic processes, such as nutrient competition, have more important effects on the succession of cyanobacterial communities than stochastic processes. Although *Microcystis* is predominant in some freshwater ecosystems, opportunistically dominant species, such as *Phormidium*, may also proliferate and should be considered in the daily monitoring of cyanobacterial blooms.

## Figures and Tables

**Table 1:** Cyanobacterial diversity indices based on OTUs of *cpcBA*-IGS. H′ = Shannon index, D^−1^ = Inverse of Simpson index (D), E = Simpson’s evenness index.

Sample	OTU Richness	Coverage	Chao 1 Index	H′	D^−1^	E
EHN21002	247	0.95	891	2.05	3.56	0.01
EHN21005	254	0.94	1087	2.06	3.63	0.01
EHN21008	188	0.96	737	1.06	1.57	0.01
EHN21012	188	0.96	933	1.05	1.55	0.01
EHN2D1002	252	0.94	1380	2.29	5.63	0.02
EHN2D1005	179	0.96	1209	0.55	1.15	0.01
EHN2D1008	258	0.94	1386	1.82	2.33	0.01
EHN2D1012	260	0.94	948	3.00	8.90	0.03
EHM11002	252	0.94	1060	2.21	4.28	0.02
EHM11005	259	0.94	859	1.92	2.68	0.01
EHM11008	210	0.95	1857	0.82	1.29	0.01
EHM11012	162	0.96	1416	0.67	1.21	0.01
EHM1D1002	224	0.95	924	1.95	3.13	0.01
EHM1D1005	208	0.95	1023	1.89	3.56	0.02
EHM1D1008	193	0.96	918	1.98	3.63	0.02
EHM1D1012	143	0.97	514	0.94	1.44	0.01
EHS21002	146	0.97	848	0.72	1.27	0.01
EHS21005	186	0.96	963	1.79	3.20	0.02
EHS21008	197	0.96	817	1.97	3.65	0.02
EHS21012	154	0.97	929	0.74	1.30	0.01
LSW1102	167	0.96	735	1.86	3.44	0.02
LSW1105	118	0.97	910	0.40	1.11	0.01
LSW1109	120	0.97	509	0.58	1.19	0.01
LSE1102	158	0.97	651	0.70	1.29	0.01
LSE1105	133	0.97	620	0.46	1.14	0.01
LSE1109	142	0.97	531	0.55	1.16	0.01
LSED1102	171	0.96	774	1.74	2.95	0.02
LSED1109	131	0.97	688	0.48	1.13	0.01

**Table 2:** Mantel tests for the correlations between environmental factors and cyanobacterial community compositions.

	Erhai Lake				Lushui Reservoir			
	Bray–Curtis		Jaccard		Bray–Curtis		Jaccard	
	*r*	*p*	*r*	*p*	*r*	*p*	*r*	*p*
TP	0.350	0.060	0.363	0.051	−0.227	0.667	−0.210	0.667
TN	−0.016	0.513	−0.016	0.510	0.154	0.336	0.172	0.336
TN/TP	0.495	0.009	0.505	0.007	−0.358	0.935	−0.354	0.942
NH_4_-N	−0.256	0.892	−0.264	0.903	0.201	0.233	0.231	0.233
NO_3_-N	0.142	0.195	0.138	0.206	−0.012	0.374	−0.003	0.371
WT	0.197	0.118	0.205	0.101	0.439	0.050	0.470	0.049
DO	0.287	0.100	0.28	0.096	0.559	0.061	0.590	0.060
SD	−0.063	0.573	−0.044	0.526	NA	NA	NA	NA

Note: NA, not available.

## Supplementary Materials

The following are available online at https://www.mdpi.com/1660-4601/17/17/6356/s1, Figure S1: Length distribution of high-quality sequences, Figure S2: Rarefaction curves of OTU richness (A), Chao 1 index (B), and Shannon index (C) against the number of sequences, Figure S3: Unrooted neighbor-joining tree of representative *cpcBA*-IGS sequences from 52 OTUs and reference cyanobacterial strains. Genbank accession number of each reference sequence was displayed before the species name. Bootstrap values above 50% are indicated at the nodes of the tree, Table S1: Detailed information of samples and number of robust reads, Table S2: Reverse primers used for amplification and pyrosequencing of *cpcBA*-IGS, Table S3: Water quality parameters in the two eutrophic lakes, Additional file 1: The normalized number of sequences of dominant OTUs in the samples.
